# Gallic acid production under anaerobic submerged fermentation by two bacilli strains

**DOI:** 10.1186/s12934-015-0386-2

**Published:** 2015-12-30

**Authors:** Pedro Aguilar-Zárate, Mario A. Cruz, Julio Montañez, Raúl Rodríguez-Herrera, Jorge E. Wong-Paz, Ruth E. Belmares, Cristóbal N. Aguilar

**Affiliations:** Group of Bioprocesses, Food Research Department, School of Chemistry, Universidad Autónoma de Coahuila, 25280 Saltillo, Coahuila Mexico; Department of Food Science and Food Technology, Universidad Autónoma Agraria Antonio Narro, 25315 Saltillo, Coahuila Mexico

**Keywords:** Tannase, Gallic acid, Bacterial strains, Anaerobic, Submerged fermentation, Liquid chromatography

## Abstract

**Background:**

Tannase is an enzyme that catalyses the breakdown of ester bonds in gallotannins such as tannic acid. In recent years, the interest on bacterial tannases has increased because of its wide applications. The lactic acid bacteria (LAB) plays an important role in food tannin biotransformation, it has the ability of hydrolyse tannins in ruminants intestine. The finding of tannin hydrolysis by LAB has sparked their use as tannase producer.

**Results:**

The bacterial strains used in the present work were identified as *Bacillus subtilis* AM1 and *Lactobacillus plantarum* CIR1. The maximal tannase production levels were 1400 and 1239 U/L after 32 and 36 h of fermentation respectively, for *B. subtilis* AM1 and *L*. *plantarum* CIR1. Maximum gallic acid release was 24.16 g/L for *B. subtilis* AM1 and 23.73 g/L for *L. plantarum* CIR1. HPLC analysis showed the formation of another peaks in the retention time range of 9–14 min, which could be attributed to the formation of di or tri-galloyl glucose.

**Conclusions:**

According to database, the strains were identified as *Bacillus subtilis* AM1 and *Lactobacillus plantarum* CIR1. In conclusion, both strains had the capability to produce good titres of extracellular tannase and release gallic acid.

## Background

Tannase (Tannin-acyl-hydrolase, E.C. 3.1.1.20) is an industrially important microbial enzyme. It catalyses the hydrolysis of ester and depside bonds in hydrolysable tannins such as tannic acid. Tannase is used in the beverage industries to remove chill haze formation in beer and wine [1, 2]. Additionally, it widely applied to reduce the antinutritional effects of poultry and animal feed along with food detanification and industrial effluent treatment [3–5]. This enzyme is also used in the manufacturing of instant tea and gallic acid, a substrate for the antioxidant propyl gallate production and trimethoprim synthesis [6, 7].

The worldwide annual demand of gallic acid is 8000 tonnes approximately [8] and the natural occurrence is restricted. Nowadays, gallic acid is industrially produced by acid hydrolysis of natural occurring gallotanins. Due to the high costs, low yield of desired product and production of large toxic effluent by acid hydrolysis, an enzyme based eco-friendly technology for gallic acid production is urgently required. Microorganisms are an alternative to the gallic acid production; because they have the ability to degrade tannic acid by producing tannase [9–11].

Most of the reported tannase producing microorganisms are fungi [1], such as *Aspergilii, Penicilii, Fusaria,* and *Trichoderma* [12]. For industrial purposes, a major problem in the utilization of fungal strains is their degradation rate is relatively slow [13]. In case of bacteria, few strains are known to be tannase producer. The tannase producing bacteria include certain species of *Bacillus*, *Corynebacterium* sp., *Lactobacillus* sp., *Serratia* sp. [14], *Enterococcus* [15]*, Streptococcus* [16]*, Pseudomonas* [17]. In this context, literature reports related to tannase production by bacteria is limited in comparison to fungal tannase; therefore the development of studies for tannase production and gallic acid synthesis is crucial to diminish production costs. The aim of the present work was to produce tannase enzyme under anaerobic conditions by two recently isolated bacilli strains. Also, the bioconversion of tannic acid to gallic acid by the enzyme produced was evaluated.

## Results and discussion

### Microorganism identification

**Fig. 1:**
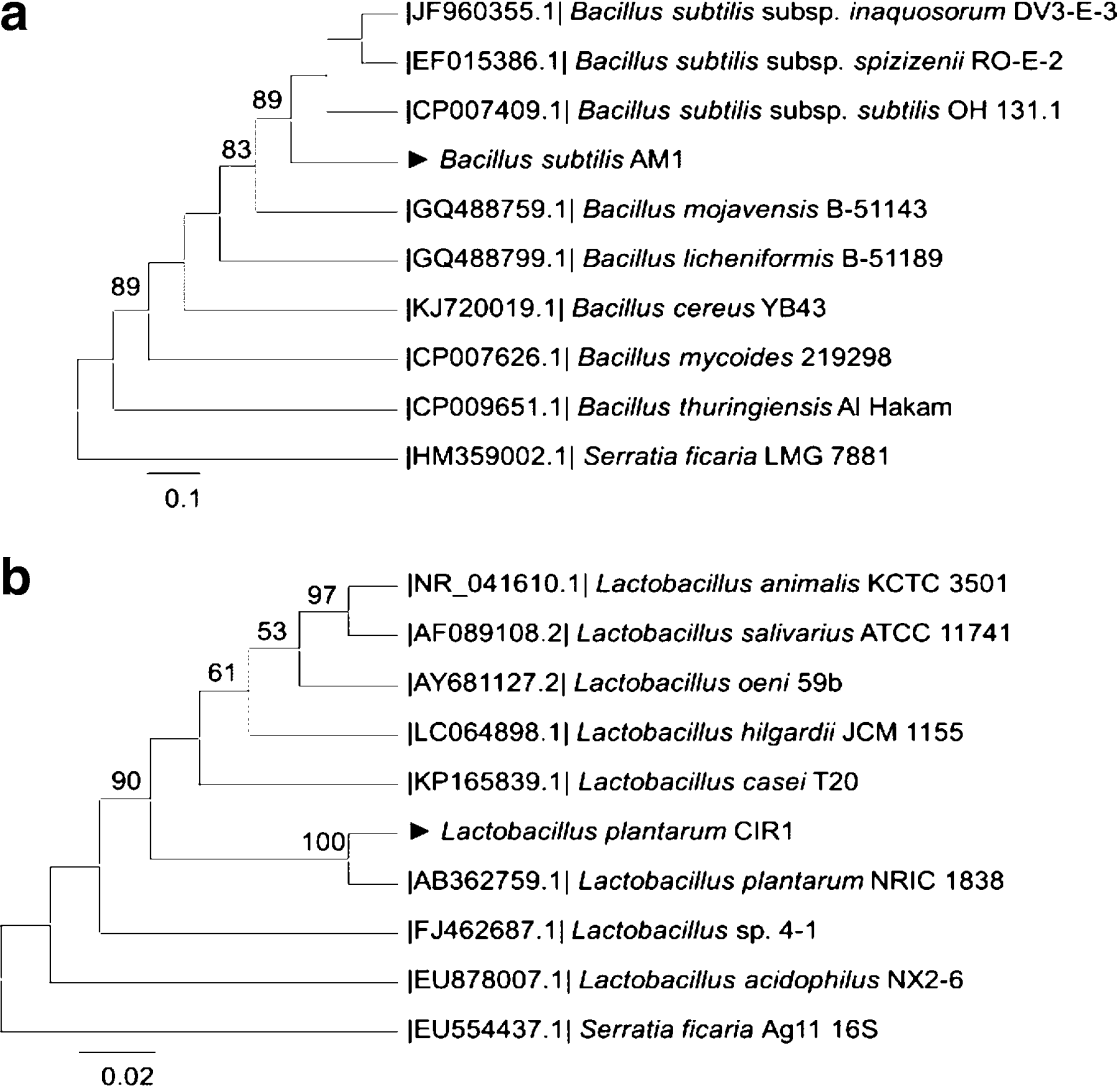
Neighbor-Joining consensus tree of **a** *Bacillus subtilis* AM1 and **b** *Lactobacillus plantarum* CIR1. The identification was based on the RNA polymerase subunit beta (rpoβ) gene for *B. subtilis* AM1 and the 16S RNA gene for *Lactobacillus plantarum* CIR1. The numbers over branches represent bootstrap confidence values (%) based on 1000 replicates. Values below 50 % are not shown. The *scale bar* denotes the nucleotide substitutions per sequence. The Genbank accessions are shown in parenthesis

Many microorganisms, including bacteria as *Lactobacillus plantarum* [14], fungi such as *Aspergillus niger* [2], and yeast as *Candida* sp. [4], have been reported as tannase producers. Extensive screening studies have been conducted to find potent cultures with high tannase production capacity. In this study two bacterial strains were used to analyze their capability to produce extracellular tannase. PCR was used to amplify the 16S rRNA gene from both bacterial genomes. However, the *rpoB* gene was amplified only from the AM1 strain. This is due to *rpoB* gene is amplified from the *Bacilli* genus. Figure 1 shows the Neighbor-Joining analysis for both bacteria. In the phylogram for *Bacillus* species (Fig. 1a) it was observed that AM1 strain was clustered with *Bacillus subtilis* species and showed an 89 % of identity to the three tested *Bacillus subtilis* strains. Figure 1b shows the phylogram for the CIR1 strain. Clearly, it can be noted that CIR1 strain was closely related to *Lactobacillus plantarum* NRIC 1838 (100 % of identity) and formed a separated clade. Both strains were compared with *Serratia ficaria* strains in order to separate the clade and demonstrate the genetic differences among species despite being the same compared gene. The taninolitic bacteria were identified as *Bacillus subtillis* AM1 and *Lactobacillus plantarum* CIR1, according to the *rpoB* and 16S rRNA gene comparison at NCBI and the comparison by the Neighbor-Joining analysis. *Bacillus subtilis* and *Lactobacillus plantarum* strains both have been reported as tannase producer [14, 18, 19, 20, 21, 22]. However, there are scarce reports related to the anaerobic production of tannase and gallic acid biosynthesis by microorganisms.

### Batch fermentation and production of tannase

**Fig. 2:**
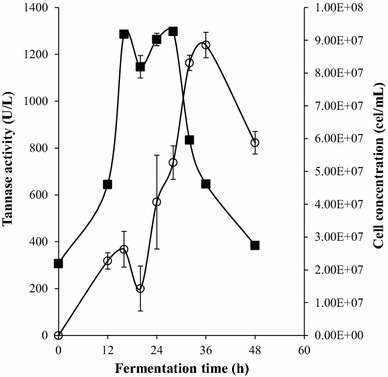
Batch fermentation by *L. plantarum* CIR1. Profile of tannase activity (*open circle*) and cell concentration (*filled square*)

The kinetic production of tannase by the strain *L. plantarum* CIR1 showed that the microorganism starts its growth exponential phase after 12 h of fermentation, while the maximum growth was at 28 h of fermentation (Fig. 2). According to this figure, a fast growth of the strain CIR1 was observed, indicating that the bacteria was metabolizing tannic acid for growth and synthesis of tannase. However, a slight diminution in growth-rate was observed at 18 h of fermentation, being this effect directly associated with a decrease in the enzyme activity. The tannase activity started at 12 h of fermentation obtaining the maximum (1239 U/L) at 36 h. Many authors have reported tannase titres higher than 5000 U/L for *L. plantarum* [18–21], however they used agitation speed on the batch and higher volumes that can induce stress in the microorganism to increase tannase activity.

**Fig. 3:**
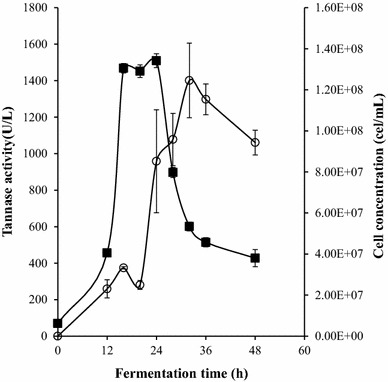
Batch fermentation by *B. subtilis* CIR1. Profile of tannase activity (*open circle*) and cell concentration (*filled square*)

Tannase activity for *B. subtilis* AM1 was initially detected at 12 h of fermentation (Fig. 3) and reached a peak at 32 h (1400 U/L). Few works employing bacilli strains to produce tannase have been reported [12, 23, 24]. Results on tannase activity by *Bacillus* strains are contrasting. In the present work, a considerable activity of tannase was obtained from *B. subtilis* AM1 compared with the tannase activity (362 U/L) of *B. licheniformis* KBR6 [23] but, was lower than the reported for *B. subtilis* PAB2 (10,690 U/L) [22]. The highest production of bacterial tannase has been reported on *B. sphaericus* (16,540 U/L) [12].

Microorganism exponential growth was observed between 12 and 16 h of culture, while the maximum growth was reached at 28 h decreasing after that time. Retardation in anaerobic growth of *B. subtilis* was observed by Hoffman et al. [25]. They conclude that anaerobic conditions caused that adaptation phases has an unpredictable duration.

Growth studies on 25 mL flask and anaerobic conditions for both strains, indicated that the production of extracellular tannase reached the maximum production peak after the late stationary phase, contrary to many reports of maximum production in that was observed in the exponential phase of growth [12, 19]. Mondal et al. [26] reported that the maximum production of extracellular tannase occurred at the stationary phase in *Bacillus cereus*. Similar pattern was observed in *Serratia ficaria* intracellular tannase, which reaches the maximum tannase production in the late stationary phase [27].

The better adaptation of AM1 strain to the growing conditions was reflected in high productivity enzyme. Tannase productivity for *B. subtilis* AM1was 43.80 U/L/h and are 1.3 fold-higher than the enzyme productivity of *L. plantarum* CIR1. Despite of *Lactobacillus* sp. has been the most reported bacterial tannase-producer strain [11], in literature the higher values of tannase productivity were obtained using *Bacillus sphaericus* [11] and *Bacillus subtilis* [22]. However, *Lactobacillus* sp. have potential applications in degradation of food tannins [28].

### Bioconversion of tannic acid to gallic acid

**Fig. 4:**
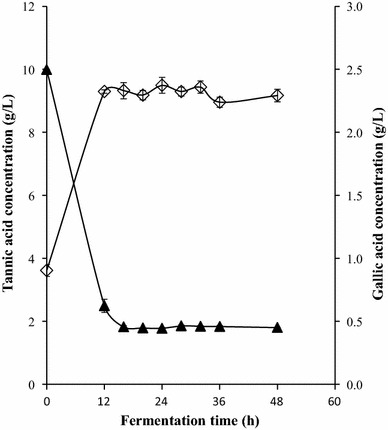
Bioconversion process by *L. plantarum* CIR1. Concentration of residual tannic acid (*filled triangle*) and gallic acid released (*unfilled diamond*)

**Fig. 5:**
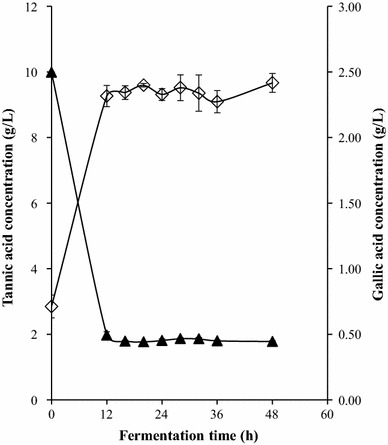
Bioconversion process by *B. subtillis* AM1. Concentrations of residual tannic acid (*filled triangle*) and gallic acid released (*unfilled diamond*)

Bioconversion profile from tannic acid to gallic acid for both strains is shown in Figs. 4 and 5. High concentration of substrate was consumed by the two strains in the first 12 h of growth while at the same time the maximum gallic acid production was 2.416 and 2.373 g/L of medium for *B. subtilis* AM1 and *L. plantarum* CIR1 respectively. The bioconversion for both strains are lower compared with similar reports in literature, however, conditions were different. In a previous work related to *L. plantarum* CIR1 the maximal productivity of gallic acid was 8.63 g/L after 24 h of culture using a gas-lift bioreactor and optimized culture conditions [18]. Jana et al. [22] reported the releasing of 6.45 g/L of gallic acid by *B. subtilis* PAB2 at 36 h of culture by optimizing the culture media.

Gallic acid synthesis keeps constant along fermentation. According to reports, it could be possible due to the enzyme gallic acid decarboxylase is not present [29]. This enzyme is used for the production of pyrogallol from gallic acid and has been reported in bacterial strains [30, 31]. The activity of gallic acid decarboxylase is limited by the presence of oxygen [32]. However, in the present work anaerobic conditions were used and the content of gallic acid showed no differences along fermentation time (Figs. 4, 5).

### HPLC analysis

**Fig. 6:**
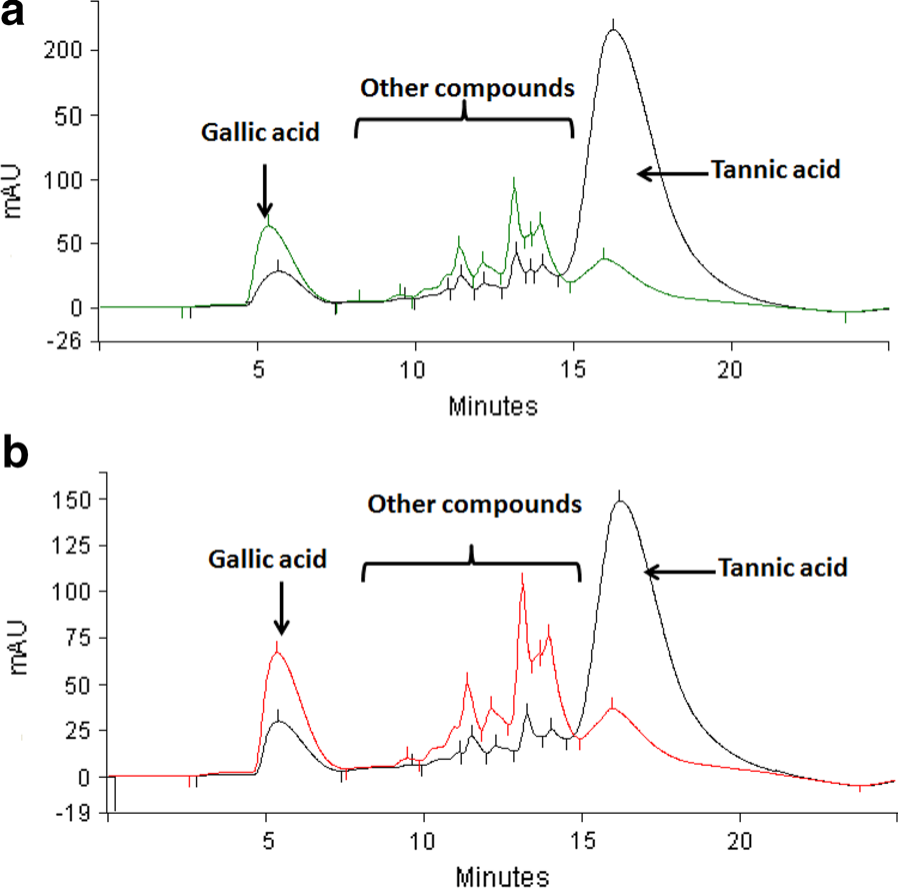
HPLC chromatograms for tannic acid hydrolysis. **a** *L. plantarum* CIR1 and **b** *B. subtilis* AM1

It is well known that HPLC analysis is very efficient in demonstrating differences in chemical constituents of samples. Figure 6 show the different peaks identified by the HPLC analysis at the 0 h and at the 48 h of fermentation in both strains. Tannic acid peak were identified at the retention time of 15.69 min, while the gallic acid peak at 6.40 min. The hydrolysis of tannic acid and the accumulation of gallic acid can be identified clearly. Formation of gallic acid at 0 h of fermentation indicates the substrate hydrolysis degree [33]. Presence of this compound was confirmed by an external standard (Sigma-Aldrich, MO, USA) in order to compare the retention time and to know the product accumulation.

In the present work, HPLC analysis revealed the formation of different peaks with different chemical intensity along the fermentation, between the retention time of 9 and 14 min (Fig. 6). These peaks could be compounds synthesized during fermentation such as di- or tri-galoyl glucose [33–35] and could be formed because of production of tannase under anaerobic stress. Hence, probably bacteria could hydrolyze that compounds after use the available carbon source producing tannase and synthesizing gallic acid.

## Conclusions

Tannase production by two bacterial strains were evaluated. Both strains have the capability to produce high titres of extracellular tannase under anaerobic conditions. Tannase production of *B. subtilis* AM1 was 1400 U/L, while for *L. plantarum* CIR1 was 1239 U/L after 32 and 36 h respectively. High concentrations of gallic acid were obtained. *B. subtilis* AM1 has the capability to release 2.416 g/L and *L. plantarum* CIR1 2.373 g/L of gallic acid. Two compounds different to gallic acid were released during the fermentation process and were identified in the HPLC analysis as two unknown peaks that could be di- or tri-galoyl glucose resulted from the partial hydrolysis of tannic acid by the action of tannase.

## Methods

### Microorganism and inoculum preparation

Two bacterial strains were obtained from the microbial collection of the Chemical Engineering Department, University of Coahuila, and were identified as AM1 and CIR1. Both strains were maintained at 35 °C, during 48 h, using own elaborate MRS agar (Man-Rogosa-Sharpe) containing (g/L): beef extract, 5; peptone, 10; yeast extract, 5; dextrose, 20; K_2_HPO_4_, 2; sodium citrate, 2; sodium acetate, 5; MgSO_4_·7H_2_O, 0.1; agar, 1.2; and Tween 80, 1. Inoculum was prepared by propagating the cryo-preserved strains in 20 mL sterile seed medium (composition same as maintenance medium excluding agar) in 25 mL flask for 24 h at 35 °C (Fisher Isotemp^®^ Incubator Senior Model, USA) under anaerobic conditions. Incubation was carried out at 35 °C for 48 h.

### Identification of microorganisms

Identification was carried out on the basis of the 16S rRNA [36] and the gene *rpoB* [37] using the primers forward (16SF 5′-AGGAGGTGATCCAACCGCA-3′; *rpoB* 5′-tcgtattctaaccatgcgcc-3′) and reverse (16SR 5′-AACTGGAGGAAGGTGGGGAT-3′; *rpoB* 5′-GCGAAGTGTTAGAATTACC-3′). The amplification was carried out in a thermal cycler with 30 µL volume containing 3 µL of 10× buffer, 10 mM of each dNTP, 2.4 µL each primer, 5 U/µL of *Taq* DNA polymerase and 80-100 ng of cDNA. Genomic DNA was isolated and quantified by standard methods using spectrophotometer Epoch Microplate Spectrophotometer™ (BioTek Instrument, Winooski, Vermont, USA). The PCR was performed in a P×2 Thermal Cycler (Thermo Electron Corporation, California, USA) with the following constituents in a program covering initial denaturation at 95 °C for 5 min; followed by 35 cycles of 1 min each at 95 °C (denaturation), 53 °C (*rpoB*) or 54 °C (16S) (alignment) and 72 °C (elongation); then an extension temperature (5 min at 72 °C) was programmed; finally the reaction was stopped at 4 °C. PCR products were electrophoresed in 1.5 % agarose gel, purified (Kit Wizard^®^ SV Gel and PCR Clean-up System, USA) and subjected to sequencing reaction. The sequence obtained was characterized by BLAST at NCBI to find out homologues with the sequences already available. The sequences were aligned using MAFFT V6 online server (http://mafft.cbrc.jp/alingment). Phylogenetic and molecular evolutionary analysis were conducted using MEGA version 6 [38] by neighbor-joining analysis of Kimura-2 parameter distance estimates. The robustness of the tree was determined by bootstrap analysis (1000 replicates).

### Batch fermentation

Fermentation was conducted in 25 mL anaerobic flasks containing 20 mL of modified medium Czapek-Dox composed of (g/L): FeSO_4_·7H_2_O, 0.01; NaNO_3_, 3; K_2_HPO_4_, 1; MgSO_4_·7H_2_O, 0.5; KCl, 0.5; and tannic acid 1 %. The production medium was adjusted to the initial pH of 6 using 1 M NaOH or 1 N HCl and sterilized (121 °C for 15 min). Culture medium was inoculated using 1 % of inoculum and the anaerobic condition was given using nitrogen to replace the oxygen. The flasks were incubated at 30 °C for the fermentation period of 48 h. Samples were withdrawn at 4 h interval after the first 12 h of fermentation when tannase activity started. The cells produced were counted using a Neubauer chamber. Then the cells were separated from the medium by centrifugation at 10,000 rpm for 15 min. The clarified supernatant was used for the analysis of tannase activity, gallic acid synthesis and tannic acid degradation.

### Tannase activity assay

The tannase activity was evaluated by a spectrophotometric method [39]. The method is based on the formation of chromogen between gallic acid (released by the action of tannase on methyl gallate) and rhodanine (2-thio-4-ketothiazolidine). For determine the tannase activity 4 solutions were prepared: citrate buffer (50 mM, pH 5), methyl gallate (0.01 M in citrate buffer 50 mM, pH 5), rhodanine (0.667 % w/v in methanol) and KOH (0.5 N). Tannase assay procedure includes the addition of 0.25 mL of crude enzyme to the same volume of methyl gallate. This was followed by the addition of 0.30 mL of rhodanine and 0.20 mL of potassium hydroxide solution with incubation at 30 °C for 5 min after each addition. Reaction was diluted with 4 mL of distilled water and again incubated at 30 °C for 10 min. The color formation was read at 520 nm using a spectrophotometer VELAB VE-5600UV (D.F., México). Tubes for blank and control were used simultaneously for each sample. One unit of tannase was defined as the amount of enzyme able to release one µmol of gallic acid formed per minute under assay conditions (temperature and time).

### HPLC analysis of gallic acid synthesis and substrate degradation

HPLC analysis was carried out according to Chávez-González et al. [33]. The clarified supernatant was filtered through a 0.45 µm membrane and subjected to analysis on an HPLC system (Varian ProStar 3300, Varian, USA) with a Star800 Photo Diode Array detector. Separation was carried out using an Octadecylsilane column (Pursuit XRs 5 C18 5 µm × 150 mm × 4.6 mm) and a mobile three-phase gradient system [A: methanol, B: acetonitrile, C: acetic acid (3 %)] at room temperature and a flow rate of 1 mL min^−1^ with an injection volume of 10 µL. Detection was carried out at 280 nm. Standard solutions (10 g/L) of tannic acid and gallic acid (Sigma-Aldrich, USA) were analyzed to compare the results.

### Statistical analysis

All experimental data were carried out in triplicate, mean values and standard deviations were calculated. Software Microsoft Excel 2007 (Microsoft Corporation, Redmond, WA, USA) was used to plot the experimental data.
